# Developmental Stage-Dependent Changes in Mitochondrial Function in the Brain of Offspring Following Prenatal Maternal Immune Activation

**DOI:** 10.3390/ijms24087243

**Published:** 2023-04-14

**Authors:** Magdalena Cieślik, Aleksandra Zawadzka, Grzegorz A. Czapski, Anna Wilkaniec, Agata Adamczyk

**Affiliations:** Department of Cellular Signalling, Mossakowski Medical Research Institute, Polish Academy of Sciences, ul. Pawińskiego 5, 02-106 Warsaw, Poland; azawadzka@imdik.pan.pl (A.Z.); gczapski@imdik.pan.pl (G.A.C.); awilkaniec@imdik.pan.pl (A.W.); aadamczyk@imdik.pan.pl (A.A.)

**Keywords:** maternal immune activation, neurodevelopmental disorders, autism, animal model, mitochondria, ROS, NADPH oxidase

## Abstract

Maternal immune activation (MIA) is an important risk factor for neurodevelopmental disorders such as autism. The aim of the current study was to investigate the development-dependent changes in the mitochondrial function of MIA-exposed offspring, which may contribute to autism-like deficits. MIA was evoked by the single intraperitoneal administration of lipopolysaccharide to pregnant rats at gestation day 9.5, and several aspects of mitochondrial function in fetuses and in the brains of seven-day-old pups and adolescent offspring were analyzed along with oxidative stress parameters measurement. It was found that MIA significantly increased the activity of NADPH oxidase (NOX), an enzyme generating reactive oxygen species (ROS) in the fetuses and in the brain of seven-day-old pups, but not in the adolescent offspring. Although a lower mitochondrial membrane potential accompanied by a decreased ATP level was already observed in the fetuses and in the brain of seven-day-old pups, persistent alterations of ROS, mitochondrial membrane depolarization, and lower ATP generation with concomitant electron transport chain complexes downregulation were observed only in the adolescent offspring. We suggest that ROS observed in infancy are most likely of a NOX activity origin, whereas in adolescence, ROS are produced by damaged mitochondria. The accumulation of dysfunctional mitochondria leads to the intense release of free radicals that trigger oxidative stress and neuroinflammation, resulting in an interlinked vicious cascade.

## 1. Introduction

Autism spectrum disorders (ASD) are a diverse group of neurodevelopmental disorders characterized by persistent challenges with social communication and social interaction, and by the presence of restricted, repetitive patterns of activities and behaviors [1]. According to epidemiological studies, it is estimated that the number of children suffering from ASD ranges from 0.19/1000 to 18.6/1000, based on various surveys and diagnostic criteria [2,3]. The biological basis and etiology of ASD are unknown, but they most likely involve an interplay between genetic and environmental influences [4]. One of the environmental factors that may detrimentally affect neurodevelopment is prenatal exposure to bacterial or viral infection, which has been implicated in the increased risk of ASD and schizophrenia by several large epidemiological studies [5,6,7,8]. This said risk was largely dependent on the gestational timing of exposure, the type of infectious agent, and, most importantly, the intensity of the maternal immune response [6]. In support of the epidemiological data, animal studies have revealed that maternal immune activation (MIA) is a profound risk for behavioral and neurochemical alterations in the progeny [6,9]; they also showed several underlying mechanisms, including cytokine imbalance (interleukin (IL)-17a [10,11,12] and IL-6 [7,13,14]) and the overactivation of microglia [15,16,17] in the brain. The consequences of MIA also include increased levels of oxidative stress, redox dysregulation, and mitochondrial dysfunction [18,19].

Mitochondria, membrane-bound organelles, are the mainstay of cells’ energy production. Under physiological conditions, mitochondria use oxidative phosphorylation (OXPHOS), a complicated reaction powered by the proton gradient across the mitochondrial inner membrane, to generate more than 95% of neuronal adenosine triphosphate (ATP), the main molecule for storing and transferring energy [20]. However, the role of mitochondria is not limited solely to energy production, as those organelles are crucial for a plethora of cellular functions, playing an important part in calcium homeostasis, thermoregulation, and cellular signaling [21]. They are also considered an integral part of several metabolic pathways, including steroid synthesis and programmed cell death. Moreover, mitochondria are key players in reactive oxygen species (ROS) generation. Even though ROS are no longer recognized as just harmful by-products, but also as an important part of the cellular signaling system [22], their overproduction is still considered disadvantageous, as they can lead to oxidative stress [23,24]. Although there are many different sources of ROS in the cell, such as endoplasmic reticulum; peroxisomes; and the activity of oxidases, such as xanthine oxidase (XO), nitric oxide synthase (NOS), and NADPH oxidase (NOX) [25,26], the mitochondria are still considered to be the main ROS generators. Therefore, mitochondrial ROS (mtROS) are constantly attracting scientific attention, especially in the brain, as this organ has a massive energy demand and is very susceptible to oxidative stress [27]. mtROS are also interesting in the context of the immune system and inflammation, as they can directly contribute to inflammatory cytokine production and innate immune responses [28], through the activation of RIG-I-like receptors (RLRs), inflammasomes, and mitogen-activated protein kinases (MAPK) [26,28,29]. Importantly, while being a major source of ROS, the mitochondria seem to be predominantly vulnerable to high levels of oxidative stress, which causes significant mitochondrial damage [30]. Mitochondrial DNA is particularly susceptible to oxidative stress, resulting in numerous mutations aggravating the disturbance of these organelles [30,31]. The abnormal functioning of the mitochondria may adversely affect cellular physiology, which can be especially dangerous during neurodevelopment. Mitochondrial dysfunction has the most impact during mid-fetal development and makes the developing brain more susceptible to environmental hits, causing changes that potentially lead to ASD [32].

A growing body of evidence suggests different mitochondrial-related disturbances in ASD patients. Several research works have demonstrated a disruption in brain energy metabolism and lactic acidosis in ASD, which are a result of mitochondrial oxidative phosphorylation dysfunction in neuronal cells [33]. Brain region-specific deficits of mitochondrial electron transport chain (ETC) complexes, oxidative stress, and glutathione redox imbalance also have been reported in autistic patients [34,35,36]. Other studies have noted decreases in the activity of non-ETC mitochondrial enzymes, such as aconitase or pyruvate dehydrogenase [37,38]. Moreover, it seems that individuals with ASD share other clinical abnormalities commonly associated with mitochondrial dysfunction such as developmental delays, ataxia, muscle weakness, and endocrine abnormalities [39,40]. What is more, it has been demonstrated that mutations in mitochondrial DNA are more common in children diagnosed with ASD than in healthy members of their families [41,42]. A large population-based study estimated that the prevalence of mitochondrial disease in ASD is 7.2% [43]. Mitochondrial disorders may also be associated with genetic syndromes that include ASD features, such as Rett syndrome [40].

As mentioned above, prenatal activation of the maternal immune system has been connected with both increased risk of ASD and with mitochondrial dysfunction. It has been demonstrated that the mitochondria have a noteworthy relationship with the immune system, as they can impact the immune response and vice versa [28]. Mitochondria are vulnerable to high levels of pro-inflammatory cytokines [44,45] and inflammatory factors released by activated glial cells could trigger an intracellular cascade, which regulates mitochondrial metabolism and function [46]. On the other hand, mitochondrial disturbance results in the release of molecules such as mitochondrial DNA, transcription factor A (TFAM), and cardiolipin, among others, which are sensed as damage-associated molecular patterns (DAMPs) recognized by immune receptors of microglia. Therefore, mitochondrial-derived DAMPs may be involved in the activation of pro-inflammatory processes and the initiation of mitochondria-dependent inflammation, so called “mitoflammation” [47]. Nevertheless, the importance of MIA-evoked mitochondrial alterations in neurodevelopmental disorders remains unclear, and it is still uncertain whether mitochondrial damage is the cause or the effect of MIA-related disorders.

Here, we investigated several aspects of mitochondrial functioning throughout brain development, first confirming the presence of MIA in fetal life, and later analyzing MIA’s influence on offspring brains in early childhood and adolescence. We observed that MIA resulted in high levels of oxidative stress and evoked perturbations in mitochondrial functioning, such as a decrease in the mitochondrial membrane potential and the production of ATP. Importantly, the observed changes were age-related, as the greatest disruptions were detected in the brain of adolescent offspring, including disturbances in the level and activity of the respiratory chain proteins, as well as those regulating the mitochondrial dynamics.

## 2. Results

### 2.1. Maternal Immune Activation Induces Free Radical Generation and Mitochondrial Dysfunction in Fetuses 24 h after the Administration of LPS to a Pregnant Mother

**Figure 1:**
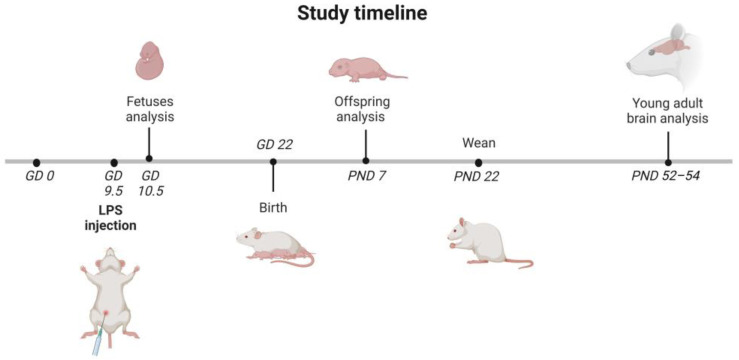
Timeline of the study. LPS (100 µg/kg b.w.) was injected intraperitoneally (i.p.) into pregnant rats at gestation day 9.5 (GD 9.5), and the control animals received a single i.p. dose of solvent (sterile 0.9% NaCl). Some of the pregnant mothers were sacrificed 24 h after the LPS administration (GD 10.5). The fetuses were collected for further research. The other dams were allowed to give birth and nurture offspring under normal conditions. The day of birth was recorded as postnatal day (PND) 1. On PND 7, each litter was equalized to 10 (both male and female) and the eliminated pups were sacrificed, their brains were removed, and they were taken for further analysis. On PND 22 to 23, rat pups were separated and housed in groups of 3 or 4. Adolescent males were sacrificed at PND 52–54, their brains were removed, and the cerebral cortex was isolated for further analysis. The figure was created with BioRender.com.

Our rat model of MIA involved the use of LPS applied to pregnant dams at GD 9.5 (Figure 1).

**Figure 2:**
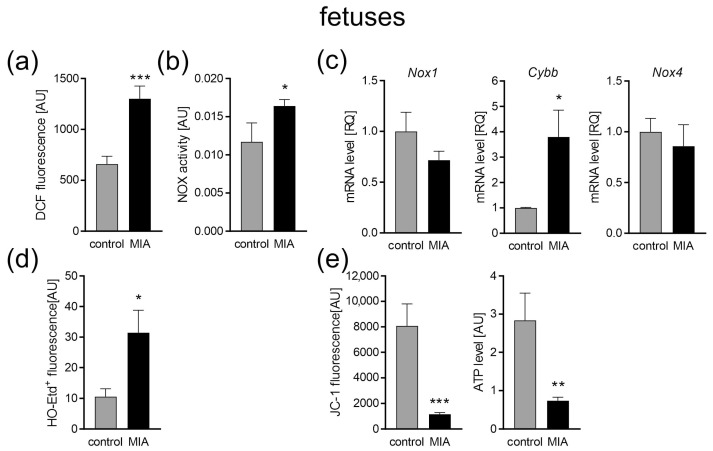
The effect of MIA on oxidative stress and mitochondrial function in fetuses. Twenty-four hours after maternal LPS administration, rat fetuses were sacrificed and whole brains were collected. (**a**) The level of ROS was determined with a DCFH-DA probe (n = 8). (**b**) The activity of NADPH oxidase (NOX) was determined by the initial rate of inhibitable ferricytochrome c reduction (n = 5 and 7). (**c**) The mRNA levels of NOX subunits *Nox1*, *Cybb*, and *Nox4* were determined using real-time PCR and were calculated by the ΔΔCt method with *Actb* (β-actin) as a reference gene (n = 6 and 5). (**d**) The generation of superoxide radicals was determined by the fluorimetric method using DHE (n = 4). (**e**) The mitochondrial membrane potential was determined by the fluorometric method using JC-1 and the mitochondrial ATP level was determined using the bioluminescence assay (n = 9 and 8). Data represent the mean value ± S.E.M. and were analysed using Student’s *t*-test, * *p* < 0.05, ** *p* < 0.01, *** *p* < 0.001, compared with the control group.

An LPS dose of 100 μg/kg body weight was previously shown to induce a short-term rise in oxidative stress and pro-inflammatory cytokines in the brain and blood of adult female rats [48]. In addition, we have shown that LPS-induced MIA in fetuses and placenta evoked an increase in the expression of some inflammation-related genes as well as an increase in the level of Iba1, a marker of microglia and macrophages [49]. Transient sickness behavior, including changes in water intake and body temperature, was also observed in the LPS-injected dams [17]. The present results support and extend our previous studies, as we demonstrate that LPS-evoked MIA induced significant alterations in the embryo biochemical state (Figure 2a–d) even 24 h after LPS administration. Higher oxidative stress was indicated by significantly increased levels of total reactive oxygen species (ROS) analyzed using 2′,7′-dichlorodihydrofluorescein diacetate (DCFH-DA) assay (*p* = 0.0003; Figure 2a). Because ROS-generating NADPH oxidases (NOX) have been shown to play a pathogenic role under inflammatory conditions and NOX-2 is upregulated in peripheral T cells of children with autism [50], we analyzed NOX activity in the fetuses of MIA animals. As shown in Figure 2b, fetuses isolated from MIA dames had increased activity of NADPH oxidase by about 28% compared with the controls (*p* = 0.0335). It was accompanied by an increased expression of NOX-2 gene *Cybb* (*p* = 0.0145; Figure 2c), while the expression of *Nox1* and *Nox4* remained unchanged. In addition, the generation of superoxide radical was increased in the MIA group (*p* = 0.0373; Figure 2d). Our data showed a decreased mitochondrial membrane potential (*p* = 0.0004; Figure 2e), measured by JC-1 fluorescence, and a decrease in the level of ATP in the fetal mitochondria (*p* = 0.0037; Figure 2e) 24 h after the induction of MIA.

### 2.2. The Impact of MIA on the Oxidative Stress and Function of Mitochondria in the Brain of 7-Day-Old Offspring

**Figure 3:**
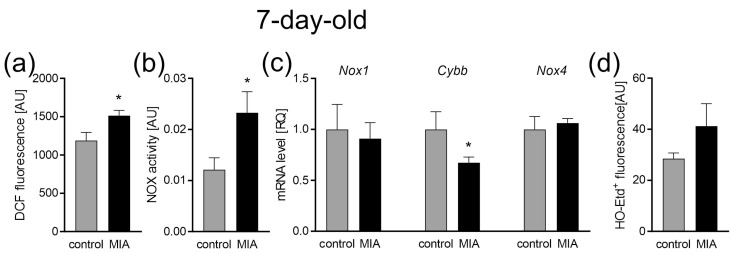
The effect of MIA on oxidative stress in the brain of 7-day-old offspring. (**a**) The level of reactive oxygen species was determined with a DCFH-DA probe (n = 8). (**b**) The level of NADPH oxidase (NOX) activity (n = 7 and 8). (**c**) The mRNA levels of NOX subunits *Nox1*, *Cybb*, and *Nox4* were determined using a real-time PCR method with *Actb* (β-actin) as a reference gene (n = 5 and 6). (**d**) The generation of superoxide radicals was determined by the fluorimetric method using DHE (n = 4). Data represent the mean value ± S.E.M. and were analysed using Student’s *t*-test, * *p* < 0.05, compared with the control group.

Our analysis demonstrated that the excessive production of ROS observed in utero also remained elevated after the birth of the offspring (*p* = 0.0117; Figure 3a). Increased NOX activity was also detected (*p* = 0.0151; Figure 3b); however, the gene expression of the two major NOX isoforms (*Nox1* and *Nox4*) remained unchanged, while the *Cybb* expression slightly decreased (*p* = 0.0422; Figure 3c). The generation of superoxide radicals was not changed (Figure 3d).

**Figure 4:**
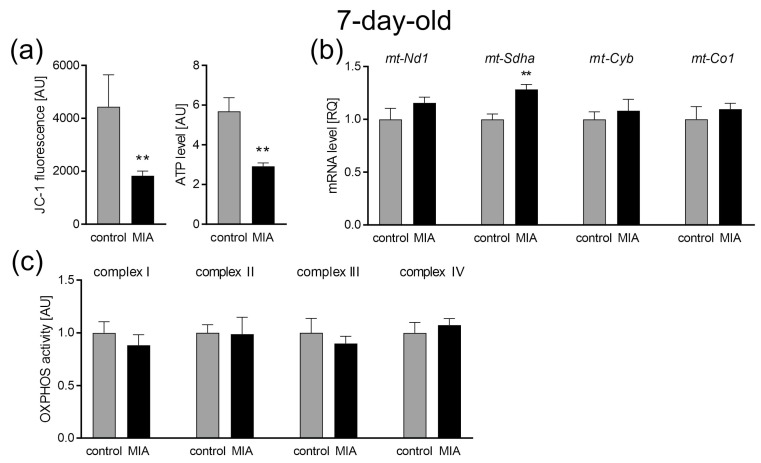
The effect of MIA on mitochondrial function in the brain of 7-day-old MIA-offspring. (**a**) Mitochondrial membrane potential was determined by the fluorometric method using JC-1 and the mitochondrial ATP level was determined using the bioluminescence assay (n = 7 and 6). (**b**) The levels of mRNA for four mitochondrial electron transport chain complexes were analysed by real-time PCR using *Actb* as a reference gene: *mt-Nd1* (n = 4 and 5), *mt-Sdha* (n = 4 and 5), *mt-Cyb* (n = 4 and 5), and *mt-Co1* (n = 4 and 5). (**c**) The activity of respiratory complexes was measured using the kinetic spectrophotometric method: complex I (n = 7 and 5), complex II (n = 9), complex III (n = 5 and 4), and complex IV (n = 5). Data represent the mean value ± S.E.M. and were analysed using Student’s *t*-test. ** *p* < 0.01, compared with the control group.

Our data also revealed changes in the parameters of the mitochondrial functioning. Both mitochondrial membrane potential (measured by JC-1 fluorescence) and ATP levels, measured in the isolated mitochondria, significantly decreased in the brain of 7-day-old offspring (*p* = 0.0055, *p* = 0.0015; Figure 4a) after prenatal exposure to LPS. Observed changes may indicate abnormalities in the functioning of the mitochondrial electron transport chain (ETC). Therefore, the gene expression and activity of the major subunits of four ETC complexes were studied. Our results revealed no change in the expression or activity of all complexes (Figure 4b,c), with the exception of Complex II (CII), for which the expression was increased (*p* = 0.0022; Figure 4b).

### 2.3. MIA Alters Gene Expression for the Fusion and Fission Proteins in the Brain of 7-Day-Old Offspring

**Figure 5:**
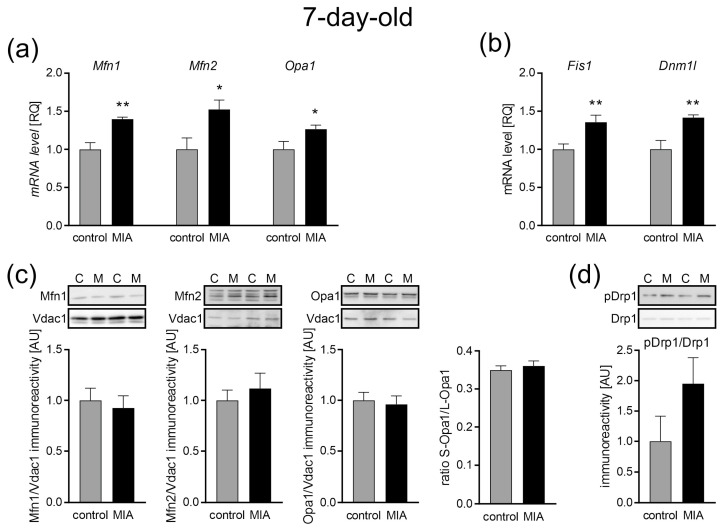
The effect of MIA on the mitochondria fusion and fission in the brain of 7-day-old offspring rats. (**a**) The level of mRNA for *Mfn1* (n = 4 and 5), *Mfn2* (n = 5), and *Opa1* (n = 5) was measured by real-time PCR with *Actb* as a reference gene. (**b**) The level of mRNA for *Fis1* (n = 5) and *Dnm1l* (n = 5) was measured by real-time PCR with *Actb* as a reference gene. (**c**) The level of immunoreactivity of Mfn1 (n = 7 and 6), Mfn2 (n = 7 and 8), Opa1, and S-Opa1/L-Opa1 ratio (n = 7 and 8) was measured using the Western blot method. Densitometric analysis was performed using normalization to the immunoreactivity of Vdac1. (**d**) The ratio of immunoreactivity of phospho-Drp1(Ser616) to total Drp1 (n = 5) was analyzed using the Western blot method and densitometric analysis. Representative immunoblots are presented. Data represent the mean value ± S.E.M. and data were analyzed using Student’s *t*-test. * *p* < 0.05, ** *p* < 0.01, compared with the control group.

As mitochondrial dynamics and morphology regulation are critical for their function, next, we analyzed the expression levels of fusion and fission proteins. Our data revealed the up-regulation of the main three genes associated with mitochondrial fusion, with increased levels of mRNA for *Mfn1*, *Mfn2*, and *Opa1* (*p* = 0.001, *p* = 0.0135, and *p* = 0.0274 respectively; Figure 5a). Similar changes were observed in the gene expression for proteins responsible for mitochondrial fission. The gene expression levels of both *Fis1* (mitochondrial fission-1 protein) and *Dnm1l* (dynamin-related protein) were elevated in the cerebral cortex of 7-day-old offspring (*p* = 0.0075 and *p* = 0.0046, respectively; Figure 5b). However, in the same experimental conditions, no change was observed in protein levels (Figure 5c), and the ratio of phospho-Drp1(Ser616) to total Drp1 also remained unchanged, although it showed a strong increasing tendency (Figure 5d).

### 2.4. No Effect of MIA on the Mitochondria Content in the Brain of 7-Day-Old Offspring

**Figure 6:**
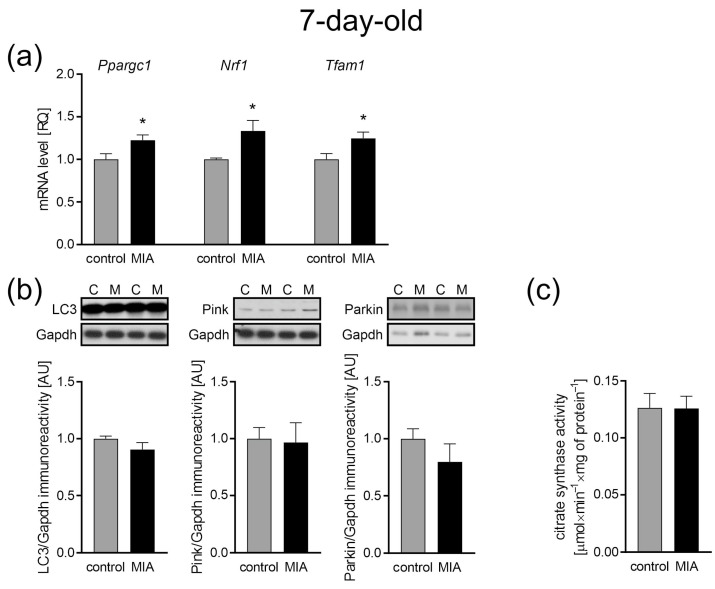
The effect of MIA on the mitochondria content, biogenesis, and autophagy processes in the brain of a 7-day-old MIA offspring rats. (**a**) The level of mRNA for the main biogenesis genes, *Ppargc1* (n = 5 and 6), *Nrf1* (n = 4 and 6), and *Tfam1* (n = 5 and 6) was measured by real-time PCR and with *Actb* as a reference gene. (**b**) The level of immunoreactivity of LC3 (n = 7), Pink (n = 7), and Parkin (n = 5 and 4) was analyzed using the Western blot method. Densitometric analysis was performed using normalization to the immunoreactivity of Gapdh. Representative immunoblots are presented. (**c**) The mitochondria content was measured by the activity of the citrate synthase (n = 8). Data represent the mean value ± S.E.M. and were analyzed using Student’s *t*-test, * *p* < 0.05 compared with the control group.

Mitochondrial biogenesis and mitochondria-selective autophagy (mitophagy) are essential for cellular adaptation in response to stress and other intracellular or environmental signals. Thus, these two opposing processes regulate mitochondrial content and influence both mitochondrial and cellular homeostasis. Here, we observed MIA-dependent changes in the gene expression of co-transcriptional regulation factors for mitochondria biogenesis in the brains of 7-day-old offspring. The gene expression level of *Ppargc* (gene for peroxisome-proliferator-activated receptor γ co-activator-1α, PGC-1α, *p* = 0.0183), *Nrf1* (nuclear respiratory factor 1, *p* = 0.0312), and *Tfam1* (mitochondrial transcription factor A, *p* = 0.019) was significantly up-regulated (Figure 6a). However, we observed no MIA-dependent changes in the immunoreactivity of the key regulator of autophagy such as microtubule-associated protein 1A/1B-light chain 3 (LC3), and mitochondrial PTEN-induced kinase 1 (Pink1) and the E3 ubiquitin ligase Parkin, team players in stress-induced mitophagy (Figure 6b). In addition, there was no difference in the citrate synthase activity (Figure 6c), a well-established marker of the mitochondria content [51,52], indicating no differences in the number of mitochondria between the control and MIA 7-day-old offspring.

### 2.5. The Impact of MIA on the Oxidative Stress and Mitochondrial Function in the Cerebral Cortex of Adolescent 54-Day-Old Offspring

**Figure 7:**
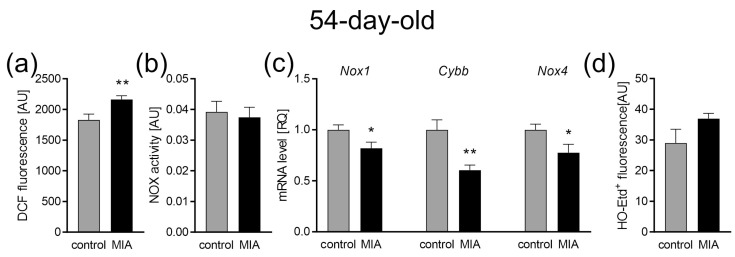
The effect of MIA on oxidative stress in the brains of 54-day-old offspring. (**a**) The level of reactive oxygen species was determined with DCFH-DA probe (n = 8). (**b**) The level of NADPH oxidase (NOX) activity (n = 9 and 6). (**c**) The mRNA level of NOX subunits *Nox1* (n = 6), *Cybb* (n = 5 and 6), and *Nox4* (n = 5 and 6) was determined using real-time PCR with *Actb* (β-actin) as a reference gene. (**d**) The generation of superoxide radicals was determined by the fluorimetric method using DHE (n = 4). Data represent the mean value ± S.E.M. and were analyzed using Student’s *t*-test. * *p* < 0.05, ** *p* < 0.01, compared with the control group.

When the MIA offspring reached adolescence (54 days of life), the oxidative stress level and main mitochondrial function were analyzed in the cerebral cortex. Our data showed that at this stage of MIA rat development, the levels of oxidative stress measured by DCF fluorescence remained increased (*p* = 0.005; Figure 7a), but were not accompanied by a shift in the activity of NADPH oxidase, as it appeared unchanged (Figure 7b). Additionally, in these conditions, the gene expression for *Nox1*, *Cybb*, and *Nox4* was downregulated (*p* = 0.0208, *p* = 0.0022, and *p* = 0.0302, respectively; Figure 7c). The generation of superoxide radicals was not changed (Figure 7d).

**Figure 8:**
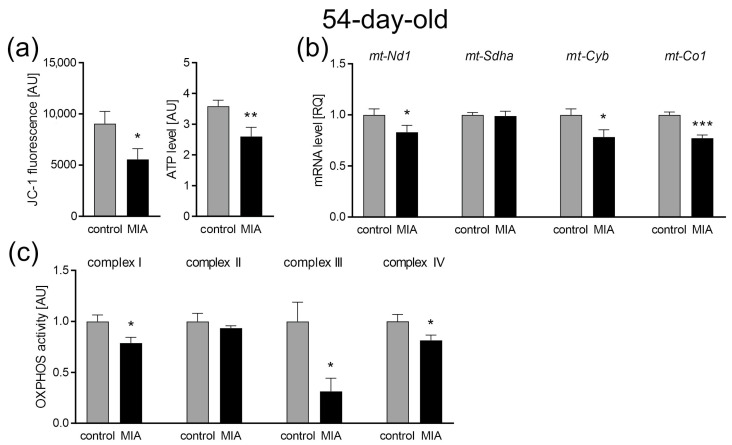
The effect of MIA on mitochondrial function in the brain of 54-day-old MIA offspring. (**a**) Mitochondrial membrane potential was determined by the fluorometric method using JC-1 (n = 5 and 9) and the mitochondrial ATP level was determined using the bioluminescence assay (n = 9 and 11). (**b**) The levels of mRNA for four mitochondrial electron transport chain complexes were analyzed by real-time PCR using *Actb* as a reference gene for *mt-Nd1* (n = 5), *mt-Sdha* (n = 5 and 4), *mt-Cyb* (n = 4), and *mt-Co1* (n = 4). (**c**) The activity of the respiratory complexes was measured using the kinetic spectrophotometric method: complex I (n = 7), complex II (n = 5 and 4), complex III (n = 5 and 4), and complex IV (n = 6 and 8). Data represent the mean value ± S.E.M. and were analyzed using Student’s *t*-test. * *p* < 0.05, ** *p* < 0.01, *** *p* < 0.001, compared with the control group.

Along with oxidative stress, a significant decrease in the mitochondrial membrane potential (JC-1 fluorescence, *p* = 0.0297; Figure 8a) and mitochondrial ATP production was indicated (*p* = 0.0091; Figure 8a). Mitochondrial disorders turned out to be much more serious in the cerebral cortex of adolescent offspring than in the 7-day-old animals as we observed a significant impairment in the ETC complexes. Our results show the downregulation of genes *mt-Nd1* (subunit of CI, *p* = 0.048), *mt-Cyb* (subunit of CIII, *p* = 0.0281), and *mt-Co1* (subunit of CIV, *p* = 0.0008) in the cerebral cortex of adolescent MIA rats (Figure 8b). Concomitant decreases in the activity of CI (*p* = 0.0151), CIII (*p* = 0.0128), and CIV (*p* = 0.0238) were observed (Figure 8c). The gene expression of the CII subunit (*Sdha*) and the activity of this complex remained unchanged (Figure 8b,c).

### 2.6. Mitochondrial Dynamics: Fission and Fusion Processes in Response to MIA in the Cerebral Cortex of Adolescent 54-Day-Old Offspring

**Figure 9:**
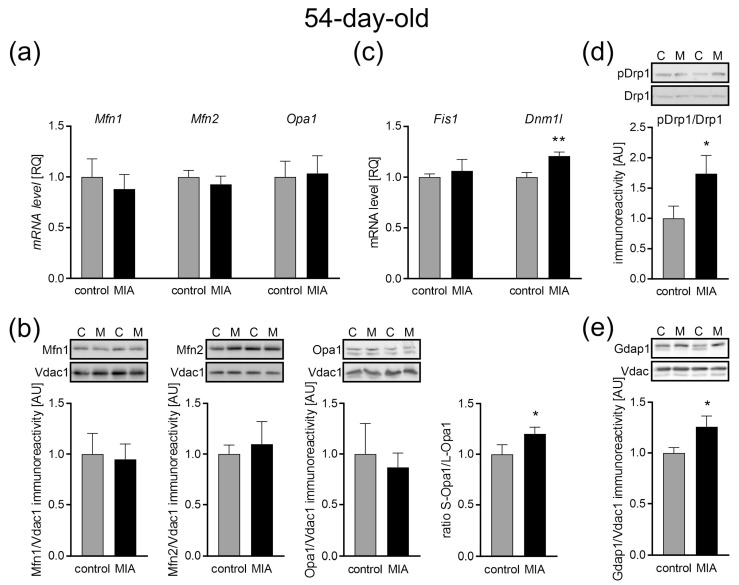
The effect of MIA on the mitochondria fusion and fission in the brain of 54-day-old offspring rats. (**a**) The level of mRNA for *Mfn1* (n = 5 and 6), *Mfn2* (n = 5), and *Opa1* (n = 6) was measured by real-time PCR with *Actb* as a reference gene. (**b**) The level of immunoreactivity of Mfn1 (n = 7 and 6), Mfn2 (n = 7), Opa1 (n = 5), and S-Opa1/L-Opa1 ratio (n = 9) was measured by the Western blot method. Densitometric analysis was performed using normalization to the immunoreactivity of Vdac1. (**c**) The level of mRNA for *Fis1* (n = 5) and *Dnm1l* (n = 4 and 5) was measured by real-time PCR with *Actb* as a reference gene. (**d**) The ratio of immunoreactivity of phospho-Drp1(Ser616) to total Drp1 (n = 4) was analyzed by the Western blot method and densitometric analysis. (**e**) The ratio of immunoreactivity of Gdap1 (n = 9 and 6) was analyzed by the Western blot method and densitometric analysis. Representative immunoblots are presented. Data represent the mean value ± S.E.M., and data were analyzed using Student’s *t*-test. * *p* < 0.05, ** *p* < 0.01, compared with the control group.

As shown in Figure 9a, the expression of fusion-related genes such as *Mfn1*, *Mfn2*, and *Opa1* was unchanged in the cerebral cortex of adolescent 54-day-old MIA offspring, compared with the control animals. Additionally, no change in protein levels was observed (Figure 9b). Inner membrane-anchored long Opa1 (L-Opa1) underwent proteolytic cleavage resulting in short Opa1 (S-Opa1) that is functionally insignificant in mitochondrial fusion. Here, we observed a slightly increased S-Opa1 to L-Opa1 ratio (*p* = 0.0489; Figure 9b), which suggests a shift into mitochondrial fragmentation and dysfunction. At the same time, the expression of the fission-associated gene *Dnm1l* was increased (*p* = 0.0052; Figure 9c), whereas the gene expression of Fis1 remained unchanged (Figure 9c). The observed increase in the Drp1 gene expression was accompanied by an increase in the ratio of phospho-Drp1(Ser616) to total Drp1 (*p* = 0.0444; Figure 9d). Ser616 phosphorylation plays an important role in the regulation of Drp1 activity and promotes its translocation from the cytosol to the mitochondrial outer membrane and enables mitochondrial fission. To further confirm the activation of mitochondrial fission in adolescent MIA rats, we analyzed the levels of the mitochondrial fission factor of the mitochondrial outer membrane (MOM), ganglioside-induced differentiation-associated protein 1 (Gdap1), which also appeared to be increased (*p* = 0.0162; Figure 9e).

### 2.7. The Influence of MIA on the Mitochondria Content in the Cerebral Cortex of Adolescent 54-Day-Old Offspring

**Figure 10:**
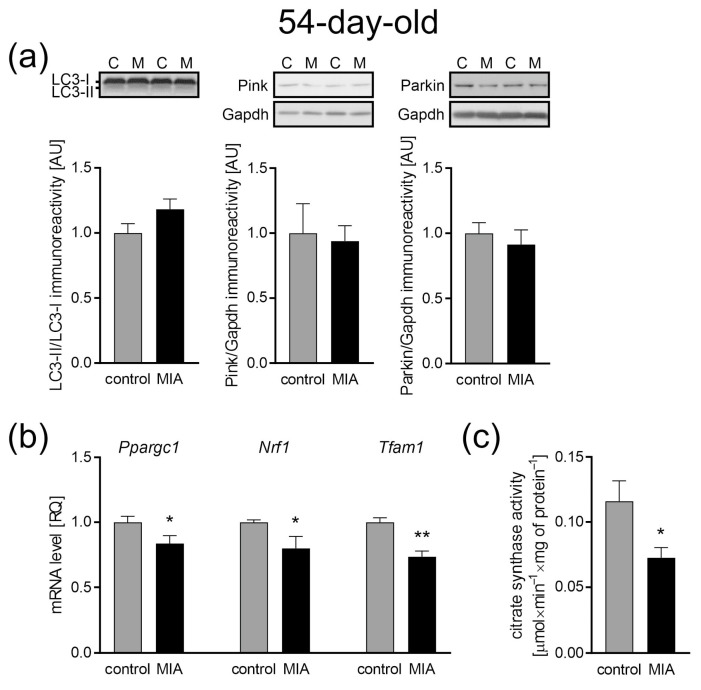
The effect of MIA on the mitochondria content, biogenesis, and autophagy processes in the brain of a 54-day-old MIA offspring rats. (**a**) The level of immunoreactivity of LC3 (n = 6 and 7), Pink (n = 6), and Parkin (n = 7 and 5) was analyzed using the Western blot method. Densitometric analysis was performed using normalization to immunoreactivity of Gapdh. Representative immunoblots are presented. (**b**) The level of mRNA for main biogenesis genes, *Ppargc1* (n = 5 and 4), *Nrf1* (n = 4), and *Tfam1* (n = 4), was measured by real-time PCR and with *Actb* as a reference gene. (**c**) The mitochondria content was measured by the activity of the citrate synthase (n = 7 and 6). Data represent the mean value ± S.E.M. and were analyzed using Student’s *t*-test, * *p* < 0.05, ** *p* < 0.01, compared with the control group.

Taking into account the abovementioned changes in mitochondrial function and dynamics, an analysis of mitochondrial biogenesis and mitophagy that regulate mitochondrial content and turnover was performed. As in the case of 7-day-old animals, our data showed no change in the LC3-II to LC3-I ratio, as well as no difference in the levels of Pink1 and Parkin in the cerebral cortex of adolescent offspring, suggesting no changes in autophagy (Figure 10a). However, key regulators of mitochondrial biogenesis were downregulated. Our research revealed a decrease in the expression of *Ppargc1* (*p* = 0.0311), *Nrf1* (*p* = 0.0385), and *Tfam* (*p* = 0.0016) (Figure 10b). Concomitantly, the data showed an MIA-dependent reduction of the citrate synthase activity, a marker of mitochondria content (*p* = 0.0189; Figure 10c).

## 3. Discussion

Our previous data demonstrated the prolonged effects of MIA on neuroinflammation and behavioral parameters in offspring [49]. We showed that MIA induced long-term changes in adolescent offspring characterized by elevated blood cytokine levels and increased pro-inflammatory cytokine expression and microglial activation. Moreover, LPS-evoked MIA resulted in autistic-like behavior in the offspring, including altered communication (reduction in emission of ultrasonic vocalizations) and impairment in social interaction (three-chamber test) [17,49]. These pathological features were accompanied by widespread ultrastructural changes in the cerebral cortex and hippocampus of MIA-exposed offspring, including alterations in the synaptic and mitochondrial structure, such as swollen mitochondria, as well as mitochondria with blurred cristae and membrane [17,49]. In this study, we focused on the source of said disturbances, questioning the connection between the observed oxidative stress and mitochondrial damage. The mitochondria, which are membrane-bound organelles, are the mainstay of cells’ energy production and are known as the center of redox processes. They can also be involved in the generation of oxidative stress [53]—a condition that occurs in the event of an imbalance between ROS production and the functioning of antioxidant mechanisms. Importantly, increased oxidative stress has been demonstrated in the brain of ASD individuals in postmortem studies [54], and redox imbalance was also observed in the blood of ASD children [55,56] and in MIA animal models [17,57].

When it comes to ROS, the mitochondria are in a difficult position, as both main ROS generators and organelles are very sensitive to high levels of free radicals. Excessive amounts of ROS harm the mitochondria by impairing lipids, proteins, and mtDNA [58]. Damage to mtDNA may lead to defective functioning of ETC complexes I and III, which can result in an increased reduction of O_2_ to O_2_^−^. Moreover, exposure to high levels of ROS may inactivate the iron−sulfur (Fe-S) centers of ETC complexes I, II, and III, resulting in decreased energy production [30]. On the other hand, the mitochondria are not the only cellular source of ROS. It seems probable that the observed oxidative stress has its origin elsewhere. Therefore, in the current study, we investigated the development-dependent changes in mitochondrial function, which may contribute to autism-like synaptic and behavioral deficits observed previously in MIA offspring.

For a better understanding of the changes that occur as a consequence of MIA, we performed the experiments at three different time points. Firstly, to confirm the occurrence of MIA, we analyzed the fetuses 24 h following intraperitoneal LPS administration to pregnant dams. Later, we collected data in the infancy (7-day-old pups) and adolescence (52 to 54-day-old rats) of MIA offspring. It allowed us to observe the development of MIA-evoked disturbances. A fair amount of research, including our previous studies, has demonstrated increased levels of pro-inflammatory cytokines in the placenta, amniotic fluid, and fetal brain of MIA-exposed animals [17,59,60,61]. It has been established that inflammation and oxidative stress go hand in hand. Chronic inflammation is more often than not accompanied by oxidative stress, whereas high levels of ROS stimulate the expression of pro-inflammatory cytokines [54]. Indeed, increased oxidative stress has been observed in amniotic fluid and fetal brain after MIA [59,62], and our results also demonstrated increased ROS, including superoxide radical, in fetuses 24 h post MIA induction. As it was mentioned above, there are different sources of ROS in the cell, other than the mitochondria. One of which is the enzyme family of NADPH oxidases (NOX), evolutionary conserved transmembrane proteins, that transport electrons from NADPH, through FAD, across the plasma membrane to O_2_, which results in the generation of O_2_^−^ [63]. Most mammals express seven NOX isoforms (NOX1, NOX2, NOX3, NOX4, NOX5, dual oxidase (DUOX)1, and DUOX2) that differ in expression in distinct tissues [64]. Few of the said isoforms are expressed in the central nervous system (CNS); NOX1, NOX2, NOX3, and NOX4 have been documented in neurons, whereas NOX1, NOX2, and NOX4 are expressed in the astrocytes and microglia [65]. It is worth mentioning that the expression of NOX2 in the microglia is low while in a resting state, with an increased expression post microglia activation [65]. The importance of NOX has been demonstrated in MIA models, even though not much data are available. It was observed that *Nox1* deficiency rescued behavioral impairments in MIA-affected offspring. In the same project, MIA up-regulated *Nox1* mRNA in the fetal brain 48 h post MIA induction [66]. Here, we demonstrated increased activity of NADPH oxidase in the fetuses 24 h post MIA, which was accompanied by an increased expression of the NOX2 gene *Cybb*, with no change in the expression of *Nox1* and *Nox4*. The upregulation of NOX2 was consistent with the results obtained in humans as NOX2 upregulation has been observed in peripheral T cells [50] as well as in blood neutrophils [67] from ASD patients. This isoform has also been connected with different neurodegenerative disorders; the inhibition of the NOX2 enzyme alleviated symptoms of Alzheimer’s disease (AD), multiple sclerosis (MS), and amyotrophic lateral sclerosis (ALS) in mice models [63], and its increased expression was detected in the post-mortem brains samples of traumatic brain injury (TBI) victims [68]. Therefore, it seems that NOX2-produced ROS might be an important factor in the generation of oxidative stress in CNS. However, we cannot say for sure that the observed increase in NOX activity was indeed only NOX2, as we measured the general activity of NADPH oxidases. We also observed signs of possible disturbance in mitochondrial functioning—decreased mitochondrial membrane potential (ΔΨm) and a decrease in ATP level in the fetal mitochondria 24 h post MIA. Proper mitochondrial function is essential for normal fetal development. The role of the mitochondria is not solely limited to energy production, as those organelles are highly involved in the signaling system and heavily influence the cells’ fate. It has been suggested that the mitochondria control the canonical developmental signaling pathways Notch, NF-κB, and Wnt, which coordinate basal cellular processes and the formation of tissues and organs, in the right place and time during prenatal life [69]. Moreover, mitochondria act as commanders of inflammation, synaptic development, and connectivity, which makes them indispensable for CNS development. Therefore, we suspect that the changes observed at this point may influence the further development of MIA offspring. Indeed, our data revealed that at least some of the observed disturbances remained until PND 7, including increased levels of ROS and increased NOX activity. However, at this time point, the expression of *Nox1* and *Nox4* remained unchanged, while *Cybb* expression was slightly decreased. In addition, an increase in superoxide generation was not as evident as in the fetuses, but some apparent tendency to increase appeared. The early postnatal period is a time of very intense changes in CNS. In mice, the cerebral metabolic rates for glucose and oxygen, energy use, and cerebral blood flow in the first 2–3 postnatal weeks (corresponding to first 5 years of life in humans) were three to seven times higher than later in life. In rats, between PND 1 and PND 21, the number of mitochondria per cell increase four-fold [70]. Any mitochondrial disturbance at this time has an effect lasting years ahead. We observed that the levels of ATP and mitochondrial membrane potential remained diminished at PND 7, which could suggest mitochondrial disturbance, but surprisingly it was not accompanied by changes in functioning of the mitochondrial ETC. We propose a few possible causes for this discrepancy. One possibility would be influence of NOX-generated ROS on ΔΨm. NOX-derived O_2_^−^ could indirectly (through PKC-ε activation) induce the opening of mitochondrial K_ATP_ channels (Mito-K_ATP_), resulting in K^+^ influx, which would lead to a decrease in ΔΨm [71]. As a result, ATP synthesis would be impaired, without disturbance to the ETC. Another explanation would be uncoupling of the ETC. The induced proton leak regulated by uncoupling proteins (UCPs) has been recognized as a protective mechanism, preventing excessive ROS production in the mitochondria—so-called “uncoupling to survive” [72]. It has been suggested that superoxide and lipid peroxidation products lead to the activation of UCPs [73,74,75]. The lack of changes in the functioning of the ETC complexes that we demonstrate here might suggest the ETC is indeed uncoupled and that it is a protective mechanism, guarding the mitochondria from harm and the entire cell from oxidative stress. However, further research is needed to fully understand these MIA-evoked mitochondrial alterations. We also analyzed mitochondrial dynamics, as fission and fusion processes are crucial for neuronal functioning, not only in mature neurons, but also during neurogenesis and neural stem cell differentiation [76]. Even though we saw an increased expression of both genes responsible for fusion and genes connected with fission, suggesting some compensation mechanism, no change in protein levels was observed. Similarly, an increased expression of mitochondrial biogenesis genes, *Ppargc1*, *Nrf1*, and *Tfam*, was observed, which might also have compensatory properties. Moreover, no change in mitochondria content or mitophagy were observed at this time point. This would mean that other than ΔΨm and ATP levels, the mitochondria do not show signs of major disturbance in 7-day-old offspring. Therefore, we suggest, that the brain mitochondria in the early postnatal period in MIA offspring life are most probably not damaged, as some possible protective mechanisms are activated. Moreover, ROS observed at this time point seems to originate from NOX activity rather than from the mitochondria.

The results obtained during the adolescence of MIA-exposed animals were quite different that those from infancy. At this time point, levels of ROS remained elevated, but were no longer accompanied by increased activity of NADPH oxidase and by the accelerated generation of superoxide radicals. This, taken together with decreases in the ΔΨm and ATP levels, pointed to the increased relevance of mitochondrial damage and mt-ROS. We suggest that at this time point, mitochondrial ETC could be the main source of free radicals and observed oxidative stress, as we saw a significant disturbance in ETC complex functioning, with a particularly dramatic decrease in complex III activity. Apart from complex I, complex III is known to be one of the main culprits behind ROS production in ETC [77]. Lower ΔΨm and ATP and a decreased expression and activity of ETC complexes suggest that the mitochondria were most likely coupled, and that observed disturbances may be a result of OXPHOS dysfunction. Moreover, changes in mRNA levels suggest a wider context of MIA-induced disturbances, as they can be caused by an abundance of factors, including ROS-induced interference with transcription [78] or even epitranscriptomic modifications [79]. It is also worth mentioning, that a decreased expression [80] and activity of mitochondrial complexes [20,81] have been observed in the brains of autistic patients.

Other aspects of mitochondrial functioning seemed to have been influenced by MIA as well, as we observed some changes in mitochondrial dynamics. The mitochondria are constantly changing structures, always remodeling the mitochondrial network in processes of fusion and fission, in order to meet the energy demands and remain in flawless shape. Mitochondrial fusion allows for sharing of mtDNA and mitochondrial proteins throughout the mitochondrial network, which protects from minor defects and promotes ATP synthesis. Fission, on the other hand, allows for quality control by isolating severe damage from the rest of the network, so it can be removed in the process of mitophagy [82]. Both are essential for cells’ well-being, but only when in balance. Different, sometimes contradictory, disturbances have been observed in ASD individuals. Tang et al. observed increased levels in fission proteins (Fis1 and Drp1) and decreased levels in fusion proteins (Mfn1, Mfn2, and Opa1) in the temporal cortex of autistic patients [20], whereas Pecorelli et al. demonstrated an increased level of Mfn1 and decreased level of Drp1 in the fibroblasts from ASD patients [83]. An analysis of the mitochondria from the oral mucosa of ASD children revealed an increased expression of not only the *MFN2* gene [84], but another group found *MFN2* to be downregulated in the anterior cingulate gyrus of ASD individuals [36]. There are few reasons these observed changes may vary. The analyses mentioned above were performed on different groups of patients (age and ethnicity) and on different tissues. Moreover, as it was mentioned before, ASD is considered an extremely diverse disorder; therefore, symptoms may be very different in various individuals. Here, we demonstrated no change in the gene expression or levels of Mfn1, Mfn2, and Opa1, except for the slightly increased S-Opa1 to L-Opa1 ratio. Fusion of the mitochondrial inner membrane was regulated by the ratio of two forms of Opa1, with excess levels of S-Opa1 adding to the downregulation of the fusion activity [85]. As for fission, we observed an increased expression of *Dnm1l* as well as an increase in the ratio of phospho-Drp1(Ser616) to total Drp1. It was accompanied by increased levels of Gdap1—the mitochondrial fission factor [86] mainly researched in the context of Charcot−Marie Tooth disease. Taken together, the obtained results indicate that the mitochondrial dynamics in our model were leaning towards fission, as expected, considering the observed disturbance in mitochondrial functioning, through decreased mitochondrial membrane potential, diminished ATP levels, and downregulation of ETC. Moreover, in our previous work, we observed ultrastructural mitochondrial changes in the cerebral cortex of MIA offspring [17]; therefore, increased fission would be beneficial, especially if it was accompanied by increased mitophagy, a process that selectively sequesters damaged or depolarized mitochondria [87]. However, current research shows no change in the LC3-II to LC3-I ratio, as well as no difference in the levels of Pink1 and Parkin. Altogether, the obtained results seemed to indicate the tendency to retain stressed or damaged mitochondria, rather than promoting their removal by mitophagy in the brains of adolescent MIA offspring. This corresponds with some recent reports, as defective mitophagy has been linked to autism and neurodevelopmental disorders in general. For example, several studies have demonstrated that *WDFY3*, *AMBRA1*, and *PARK2* gene mutations manifest as autism-like symptoms and as mitophagy dysfunction [88,89,90,91]. As damaged and dysfunctional mitochondria are most likely not being removed, and at the same time, a significant decrease in citrate synthase activity, the most commonly used marker of mitochondrial content, has been observed, which could suggest a possible disturbance in mitochondrial biogenesis. In fact, our data reveled downregulation of *Ppargc1*, *Nrf1*, and *Tfam*, which could be the reason behind the reduced amount of healthy mitochondria.

**Figure 11:**
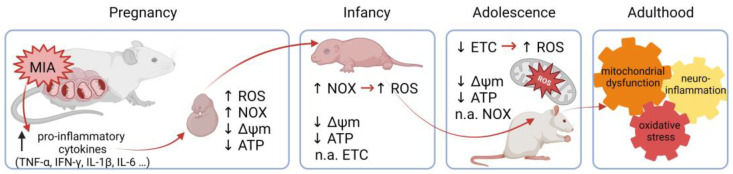
The proposed course of events. Activation of the maternal immune system induces the secretion of pro-inflammatory cytokines, affecting the developing fetus, through NOX activation and the induction of ROS generation. It is also accompanied by decreased ΔΨm and ATP levels. Increased NOX activity and ROS levels, as well as decreased ΔΨm and ATP levels, remain through infancy, but are not accompanied by a disturbance in ETC functioning, suggesting some compensatory mechanisms. High ROS levels persist until adolescence, but are no longer caused by NOX activity. An alternative source of ROS seems to be damaged mitochondria. Decreased ΔΨm and ATP levels, as well as the decreased expression and activity of ETC complexes, suggest OXPHOS dysfunction and mitochondrial damage. The accumulation of disturbed mitochondria and oxidative stress can lead to neuroinflammation and finally synaptic impairment throughout the life of an MIA individual. NOX—NADPH oxidase; ROS—reactive oxygen species; ΔΨm—mitochondrial membrane potential; ATP—adenosine triphosphate; ETC—electron transport chain; OXPHOS—oxidative phosphorylation; MIA—maternal immune activation; n.a.—not affected. The figure was created with BioRender.com.

In conclusion, our study supports and extends evidence for oxidative stress and mitochondrial damage involvement in the pathogenesis of neurodevelopmental disorders dependent on maternal inflammation during pregnancy. Although more research is needed, we suggest that MIA, through pro-inflammatory mediators, induces the NOX-derived overproduction of ROS prenatally, accompanied by decreased levels of ΔΨm and ATP, which continue in infancy, leading to mitochondrial damage later in life. We suggest that ROS observed in infancy are most likely of a NOX activity origin, whereas in adolescence, ROS are produced by damaged mitochondria (Figure 11). The accumulation of disturbed mitochondria could lead to an intense generation of free radicals and mitochondrial DAMPs, which could induce neuroinflammation and oxidative stress generation, resulting in a vicious cycle. Therefore, MIA-induced NOX-derived ROS followed by mitochondrial damage and mtROS production could play an important role in the pathogenesis of maternal infection-induced neurodevelopmental disorders.

## 4. Materials and Methods

### 4.1. Ethical Statement

All of the experiments conducted with animals were approved by the Local Ethics Committee for Animal Experimentation in Warsaw (361/2017, WAW2/021/2021) and were carried out in accordance with the EC Council Directive of 24 November 1986 (86/609/EEC), following the ARRIVE guidelines and guidelines published in the NIH Guide for the Care and Use of Laboratory Animals, and the principles presented in the “Guidelines for the Use of Animals in Neuroscience Research” by the Society for Neuroscience. Efforts were made to minimize animal suffering and to reduce the number of animals used. All manipulations were performed gently and quickly to avoid stress-induced alterations.

### 4.2. Animals and Experimental Procedure

Pregnant Wistar rats (2–3 months old, 210–250 g) were supplied by the Animal House of Mossakowski Medical Research Institute PAS (Warsaw, Poland), which operates breeding of small rodents according to the SPF standard. The MIA model was induced by a single i.p. injection of LPS of *Escherichia coli* (Sigma-Aldrich, Saint Louis, MO, USA; serotype 055:B5) at a dose of 100 µg/kg body weight on gestational day 9.5 (GD 9.5), while the controls received a single i.p. dose of solvent (sterile 0.9% NaCl) [49,92]. All of the animals were maintained under controlled conditions of temperature and humidity with a 12-h light/dark cycle. Some of the pregnant mothers were sacrificed 24 h after LPS administration (GD 10.5), and the fetuses were collected for research. The other dams were allowed to give birth and nurture offspring under normal conditions. The day of birth was recorded as postnatal day (PND) 1. On PND 7, each litter was equalized (random selection) and the number of pups was limited to 10 (both male and female). The eliminated pups were sacrificed on the same day, their brains were removed, flash-frozen whole in liquid nitrogen for further analysis, or taken immediately for biochemical analysis. On PND 22–23, the rat pups were separated and housed in groups of three or four in open polycarbonate cages in an enriched environment. To avoid hormone-related changes, only males were selected for further experimental procedures. Adolescent males were sacrificed at PND 52–54, their brains were removed, and the cerebral cortex was isolated on an ice-cooled Petri dish and flash-frozen in liquid nitrogen for further analysis.

### 4.3. Mitochondria Isolation and Determination of the Mitochondrial Membrane Potential (ΔΨm)

The isolation of intact mitochondria from the obtained tissue was performed with a Mitochondrial Isolation kit (Sigma-Aldrich, Saint Louis, MO, USA). Tissue was homogenized in an extraction buffer A (10 mM HEPES, pH 7.5; 200 mM mannitol; 70 mM sucrose; 1 mM EGTA), and centrifuged at low (600× *g* for 5 min) and high speed (11,000× *g* for 10 min). The isolated mitochondria were resuspended in 40 µL storage buffer (10 mM HEPES, pH 7.5, containing 250 mM sucrose, 1 mM ATP, 80 µM ADP, 5 mM sodium succinate, 2 mM K_2_HPO_4_, and 1 mM DTT) and used directly for JC-1 staining. JC-1 (5′,6,6′-tetrachloro-1,1′,3,3′-tetraethylbenzimidazolylcarbocyanine iodide) is accumulated in mitochondria in a potential-dependent manner, which can be detected by a fluorescence emission shift from green (527 nm) to red-orange (590 nm). The detection of the mitochondrial membrane potential was performed according to the manufacturer’s protocol, and fluorescence was read in a spectrofluorometer TECAN Infinite M1000PRO (excitation wavelength = 490 nm; slit = 5 nm, emission wavelength = 590 nm; slit = 7.2 nm). The mitochondrial membrane potential was calculated as fluorescence produced in the mitochondria suspension per milligram of mitochondrial protein and expressed as arbitrary units (AU).

### 4.4. Measurement of the Reactive Oxygen Species (ROS) Level

The level of ROS was measured using fluorescent probe 2′,7′-dichlorodihydrofluorescein diacetate (DCFH-DA) with the previously described protocol [48]. DCFH-DA is deacetylated by cellular esterases to 2′,7′-dichlorodihydrofluorescein (DCFH), which can then be oxidized to a highly fluorescent 2′,7′-dichlorofluorescein (DCF). Tissue homogenate (1% in PBS) was incubated with 10 µM DCFH-DA for 45 min at 37 °C in darkness. DCF fluorescence was measured in duplicate for each sample, using a microplate reader TECAN Infinite M1000PRO (488 nm excitation and 525 nm emission wavelengths). Additional incubation with 10 µM FeCl_2_ was performed as a positive control. The results of the fluorescence measurements were normalized to the protein concentration and presented as arbitrary units (AU).

### 4.5. Measurement of the Superoxide Radical Level

The level of superoxide radical generated in situ was determined using dihydroethidium (DHE) as a fluorogenic probe [93,94]. DHE oxidation by superoxide anion leads to the formation of fluorescent 2-hydroxyethidium (Ex 400 nm, Em 590 nm). The protocol was based on the method described previously [95]. Shortly, the tissue homogenate (1% in PBS) was mixed with pre-warmed PBS supplemented with glucose (5 mM) and DHE (20 µM). Immediately, fluorescence was measured using a microplate reader TECAN Infinite M1000PRO (400 nm excitation and 590 nm emission wavelengths) for 3 min at 37 °C. The results of the fluorescence measurements were normalized to the protein concentration and presented as arbitrary units (AU).

### 4.6. Measurement of the NOX/NADPH Oxidase (NOX) Activity

NOX activity was determined by the initial rate of inhabitable ferricytochrome c reduction, and the measurements were performed based on the methods described by Shpungin et al. [96]. Tissue samples were homogenized in ice-cold buffer (Phosphate Buffered Saline, pH 7.4, 5% homogenates). The reaction buffer contained 100 mM K,Na-phosphate buffer, 1 mM SDS, 1 mM cytochrome c, and 1 mM FAD. Before being added to the reaction buffer, cytochrome c was reduced by incubation for 20 min in darkness with DTT (100 mM). The enzyme activity in the homogenates was measured with and without the presence of the NADPH oxidase inhibitor (apocynin, 5 mM). The reaction was initiated by the addition of NADPH (2 mM). Each sample was measured on a microplate reader Multiscan GO (Thermo Scientific) in duplicate at 550 nm in a kinetic loop for 30 min (kinetic interval 1 min). The enzyme activity was calculated as the difference between measurements with and without an inhibitor in one chosen time point (same for every sample) and presented as arbitrary units (AU).

### 4.7. Determination of Mitochondrial ATP Levels

The level of mitochondrial ATP was measured using the luminescence assay ATP Determination Kit (Thermo Fisher Scientific, Waltham, MA, USA), according to the manufacturer’s protocol. The assay was based on luciferase’s absolute requirement for ATP in producing light. Each sample was measured in duplicate, using a microplate reader TECAN Infinite M1000PRO (560 nm). The ATP levels were calculated as luminescence produced in the mitochondria suspension per milligram of mitochondrial protein and were expressed as arbitrary units (AU).

### 4.8. Determination of Gene Expression (Real-Time PCR)

RNA was isolated with TRI-reagent according to the manufacturer’s protocol (Sigma-Aldrich, Saint Louis, MO, USA), and its quantity and quality were controlled by spectrophotometric analysis using a NanoDrop ND-1000 spectrophotometer (NanoDrop Technologies, Wilmington, DE, USA). Digestion of DNA was performed with DNase I according to the manufacturer’s protocol (Sigma-Aldrich, Saint Louis, MO, USA). Reverse transcription was performed with a High Capacity cDNA Reverse Transcription Kit according to the manufacturer’s instructions (Applied Biosystems, Foster City, CA, USA). The expression levels of mRNA were measured with real-time PCR, using the TaqMan Gene Expression Assays (Applied Biosystems, Foster City, CA, USA) *Nox1* (Rn00586652), *Cybb* (Rn00576710), *Nox4* (Rn00585380), *mt-Nd1* (Rn03296764_s1), *mt-Sdha* (Rn00590475_m1), *mt-Cyb* (Rn03296746_s1), *mt-Co1* (Rn03296721_s1), *Mfn1* (Rn00594496), *Mfn2* (Rn00500120), *Opa1* (Rn00592200), *Dnm1l* (Rn00586466), *Fis1* (Rn01480911), *Ppargc1* (Rn00580241), *Nrf1* (Rn01455958), *Tfam* (Rn00580051), and *Actb* (Rn 00667869_m1) as the reference gene, on an ABI PRISM 7500 apparatus. *Actb* was chosen as a reference gene, as its expression appeared not changed in comparison to the control (Supplementary Table S1). The relative changes in mRNA levels were calculated using the ΔΔCt method and expressed as RQ.

### 4.9. Citrate Synthase Activity Assay

The activity of citrate synthase in the samples was determined using a MitoCheck^®^ Citrate Synthase Activity Assay Kit (Cayman Chemical, Ann Arbor, MI, USA) according to the manufacturer’s instructions. The activity of the enzyme was determined by measuring the production of SH-CoA by monitoring the absorbance of Citrate Synthase Developing Reagent. Each sample was measured in duplicate on a microplate reader Multiscan GO (Thermo Fisher Scientific, Waltham, MA, USA) (OD = 412 nm) and the activity was expressed as the change in absorbance per minute per amount of protein.

### 4.10. Determination of Protein Level

The concentration of proteins in the samples was determined using the Pierce^TM^ BCA Protein Assay Kit (Thermo Fisher Scientific), according to the manufacturer’s protocol. Measurements were performed in duplicate, on a microplate reader Multiscan GO (Thermo Fisher Scientific) at 562 nm absorbance.

### 4.11. Immunochemical Determination of Protein Levels (Western Blot Analysis)

The immunochemical analysis of the protein level and phosphorylation status was performed using the Western blotting method under standard conditions. Tissue samples were homogenized, mixed with a Laemmli buffer, and denatured at 95 °C for 5 min. After standard SDS-PAGE separation, the proteins were transferred onto nitrocellulose membranes under standard conditions. The membranes were washed for 5 min in TBST (Tris-buffered saline with Tween 20 buffer: 100 mM Tris, 140 mM NaCl, and 0.1% Tween 20, pH 7.6), and non-specific binding was blocked for 1 h at room temperature (RT) with 5% non-fat milk solution in TBST. The blocked membranes were probed with primary antibodies: Mfn1 (ABC41, Merck), Mfn2 (M6319, Sigma-Aldrich), Opa1 (D6U6N, Cell Signaling Technology), Drp1 (ABT155, Merck), p-Drp1 (PA5-64821, Invitrogen), Gdap1 (HPA014266, Sigma-Aldrich) LC3 (L8918, Sigma-Aldrich), Pink1 (Sc-517353, Santa Cruz Biotechnology), and Parkin (Sc-32282, Santa Cruz Biotechnology). Gapdh (G9545, Sigma-Aldrich) and Vdac1 (AB10527, Merck) were used as the loading controls. The membranes were washed in TBST (×3), incubated for 60 min at RT with appropriate secondary antibodies (anti-rabbit (A0545, Sigma-Aldrich) or anti-mouse IgG (A28177, Invitrogen)), and washed again in TBST (×3). Antibodies were detected using the chemiluminescent reaction with the ECL reagent (Amersham Biosciences, Bath, UK) under standard conditions. After stripping, the membranes were re-probed. Densitometric analysis was performed with the TotalLab4 software (NonLinear Dynamics Ltd., Newcastle upon Tyne, UK).

### 4.12. Electron Transport Chain Complexes Activity Assays

The mitochondrial OXPHOS complex I activity was assessed using the Complex I Enzyme Activity Microplate Assay Kit according to the manufacturer’s protocol (Abcam). Briefly, 50 µg of protein was incubated in the wells of a microplate, which was pre-coated with the complex I capture antibody for 3 h at RT. Then, the activity of the immunocaptured complex I enzyme was determined by measuring the oxidation of NADH to NAD^+^ and the simultaneous reduction of a dye, which led to increased absorbance at 450 nm. Each sample was measured on a microplate reader Multiscan GO (Thermo Fisher Scientific) in duplicate, and the activity was calculated as a change in absorbance per minute and expressed as arbitrary units (AU).

Mitochondrial OXPHOS complex II activity was assessed using Complex II Enzyme Activity Microplate Assay Kit (Abcam), according to the manufacturer’s instructions. Microplate wells in the kit have been coated with an anti-complex II monoclonal antibody (mAb), which purifies the enzyme from a sample. The production of ubiquinol by the enzyme (by reduction of ubiquinone) was coupled to the reduction in the dye 2,6-dichlorophenolindophenol (DCPIP) and was measured as a decrease in absorbance at 600 nm. Each sample was measured in duplicate on a microplate reader Multiscan GO (Thermo Fisher Scientific), and the enzyme activity was calculated as a change in absorbance per minute and expressed as arbitrary units (AU).

The mitochondrial OXPHOS complex III activity was measured using a Mitochondrial Complex III Activity Assay Kit (Sigma-Aldrich) according to the manufacturer’s instructions. The assay was based on the reduction of cytochrome c through the activity of complex III, and the absorbance of reduced cytochrome c was measured at 550 nm. Each sample was measured on a microplate reader Multiscan GO (Thermo Fisher Scientific) in duplicate, and the activity was calculated by comparing the sample ABS 550 values (after subtraction of the background control) to the reduced cytochrome c standard curve. The enzyme activity was calculated as Δnmol cyt c × min^−1^ × mg protein^−1^ and expressed as arbitrary units (AU).

The mitochondrial OXPHOS complex IV activity was measured as an activity of the cytochrome c oxidase enzyme in the samples using a Complex IV Rodent Enzyme Activity Microplate Assay Kit (Abcam), according to the manufacturer’s instructions. The enzyme was immunocaptured within the wells of the microplate, and its activity was determined colorimetrically by following the oxidation of reduced cytochrome c, as the absorbance decreased at 550 nm. Each sample was measured in duplicate on a microplate reader Multiscan GO (Thermo Fisher Scientific), and the activity was determined by calculating the slope between two points within the linear region. The enzyme activity was calculated as ΔmOD × min^−1^ × mg protein^−1^ and was expressed as arbitrary units (AU).

### 4.13. Statistical Analysis

The results were expressed as mean values ± S.E.M. In all of the analyses, each data point is from a separate animal. The normality and equality of group variances were tested using the Shapiro–Wilk test. Differences between means were analyzed using an unpaired Student’s *t*-test. The level of statistical significance was set at *p* < 0.05. The statistical analyses were performed using GraphPad Prism version 6.0 (GraphPad Software, San Diego, CA, USA).

## Data Availability

The data presented in this study are available upon request from the corresponding author.

## Supplementary Materials

The following supporting information can be downloaded at: https://www.mdpi.com/article/10.3390/ijms24087243/s1.
