# Supplementary material for: Developmental Stage-Dependent Changes in Mitochondrial Function in the Brain of Offspring Following Prenatal Maternal Immune Activation
Source: Int J Mol Sci. 2023 Apr 14;24(8):7243. doi: 10.3390/ijms24087243 (PMC10138707; doi:10.3390/ijms24087243)
Supplement: Supplementary file 1 [file ijms-24-07243-s001.zip › ijms-2266667-supplementary.pdf]

Supplementary Table S1. The mean CT data for *Actb* obtained in the real-time PCR experiments.

	Fetuses	7-day-old	54-day-old
control	20.075	15.106	19.097
MIA	19.908	15.094	19.392
